# Efficacy and Safety of an Aflibercept Treat-and-Extend Regimen in Treatment-Naïve Patients with Macular Oedema Secondary to Central Retinal Vein Occlusion (CRVO): A Prospective 12-Month, Single-Arm, Multicentre Trial

**DOI:** 10.1155/2018/8310350

**Published:** 2018-10-14

**Authors:** Jose Garcia-Arumi, Francisco Gómez-Ulla, Navea Amparo, Enrique Cervera, Alex Fonollosa, Luis Arias, Javier Araiz, Juan Donate, Marta Suárez de Figueroa, Lucia Manzanas, Jaume Crespí, Roberto Gallego

**Affiliations:** ^1^Hospital Universitari Vall d'Hebron, Barcelona, Spain; ^2^Instituto Oftalmológico Gómez-Ulla, Complejo Hospitalario Universitario de Santiago de Compostela, A Coruña, Spain; ^3^FISABIO-Oftalmología Médica, Valencia, Spain; ^4^Hospital General de Valencia, Valencia, Spain; ^5^Hospital Universitario de Cruces, Barakaldo, Spain; ^6^Hospital Universitari de Bellvitge, Barcelona, Spain; ^7^Instituto Clínico Quirúrgico de Oftalmología, Bilbo, Bizkaia, Spain; ^8^Hospital Clínico San Carlos, Madrid, Spain; ^9^VISSUM, Madrid, Spain; ^10^Hospital Clínico Universitario de Valladolid, Valladolid, Spain; ^11^Hospital de la Santa Creu i Sant Pau, Barcelona, Spain; ^12^Unidad de Mácula, Clínica OFTALVIST, Valencia, Spain

## Abstract

**Objectives:**

To evaluate efficacy and safety of an aflibercept treat-and-extend (TAE) regimen in patients with macular oedema (MO) secondary to central retinal vein occlusion (CRVO).

**Design, Setting, and Patients:**

Phase IV, prospective, open-label, single-arm trial in 11 Spanish hospitals. Treatment-naïve patients with <6 month diagnosis of MO secondary to CRVO and best-corrected visual acuity (BCVA) of 73-24 ETDRS letters were included between 23 January 2015 and 17 March 2016.

**Intervention:**

Intravitreal aflibercept 2 mg monthly (3 months) followed by proactive individualized dosing.

**Main Outcomes:**

Mean change in BCVA after 12 months.

**Results:**

24 eyes (24 patients) were included; mean (SD) age: 62.8 (15.0) years; 54.2% male; median (IQR) time since diagnosis: 7.6 (3.0, 15.2) days. Mean BCVA scores significantly improved between baseline (56.0 (16.5)) and Month 12 (74.1 (17.6)); mean (95% CI) change: 14.8 (8.2, 21.4); *P*=0.0001. Twelve (50.0%) patients gained ≥15 ETDRS letters. Foveal thickness improved between baseline (mean: 569.4 (216.8) *µ*m) and Month 12 (mean 257.4 (48.4) *µ*m); *P* < 0.0001. At Month 12, 8.3% patients had MO. The mean (SD) number of injections: 8.3 (3.0). No treatment-related AEs were reported. Five (20.8%) patients experienced ocular AEs. Two nonocular serious AEs were reported.

**Conclusions:**

An aflibercept TAE regimen improves visual acuity in patients with MO secondary to CRVO over 12 months with good tolerability.

## 1. Introduction

Retinal vein occlusion (RVO) is thought to result from a thrombotic event or vessel wall pathology that can severely impact visual acuity [1, 2]. Macular oedema (MO) secondary to RVO is the second-most common retinal vascular disease after diabetic retinopathy and is subdivided into central retinal vein occlusion (CRVO) and branch retinal vein occlusion (BRVO) based on the location of the occlusion [3].

Central retinal vein occlusion is estimated to affect between 0.3% and 2.1% of the global population with no significant variation in prevalence due to ethnicity or gender [4]. Like BRVO, clinical features of CRVO include dilated and tortuous retinal veins, deep and superficial retinal haemorrhages, cotton wool spots, and retinal oedema [5]. However, unlike BRVO, these features are found in all quadrants of the retina in CRVO. Visual loss after CRVO commonly occurs as a result of MO, ischemia, or in more advanced stages, occurs as a result of vitreous haemorrhage or neovascularisation.

Current therapeutic options for the treatment of MO secondary to CRVO include laser photocoagulation, vascular endothelial growth factor (VEGF) inhibitors, and intraocular steroids [6]. Several recent meta-analyses have shown treatment of MO secondary to CRVO with anti-VEGF agents resulted in improved best-corrected visual acuity (BCVA) over time and fewer adverse events compared to placebo, corticosteroids, or laser photocoagulation [7–9]. An assessment of clinical evidence by the American Academy of Ophthalmology concluded that anti-VEGF pharmacotherapy was safe and effective over two years for MO secondary to CRVO [10]. Network meta-analysis of drug treatments to manage MO secondary to CRVO found no evidence of differences in efficacy between the anti-VEGF agents: ranibizumab, aflibercept, bevacizumab, or corticosteroid triamcinolone [11].

The recombinant fusion protein aflibercept (Eylea; Regeneron Pharmaceuticals), a fusion protein of key domains from human VEGF receptors 1 and 2, was approved by the US Food and Drug Administration (FDA) in 2011 and by the European Medicines Agency in 2012 for the treatment of patients with neovascular age-related macular degeneration (nAMD) [12, 13]. A higher VEGF affinity and a longer half-life compared to older anti-VEGF agents reduce the frequency of intravitreal (IVT) injections needed to maintain therapeutic effect, thus potentially reducing the clinical burden placed on patients by reducing the number of treatment visits [14–18].

The findings of the sister confirmatory phase III randomised controlled GALILEO and COPERNICUS trials showed IVT aflibercept improved 12-month visual and anatomical outcomes when administered pro re nata (PRN) following six monthly injections in patients with MO secondary to CRVO, provided that the patients were administered aflibercept following the initial event [5, 19, 20].

The treat-and-extend (TAE) regimen is a proactive individualized treatment regimen designed to maximize the benefit to risk ratio of anti-VEGF agents. It is frequently used for the treatment of nAMD [21–23]. Compared to fixed dose and PRN regimens, the TAE regimen reduces treatment burden and does not require disease reactivation for continued treatment [23]. Under this regimen, anti-VEGF agents are administered as fixed loading doses until clinical remission, followed by increasing treatment intervals until a maximal safe interval is reached (usually 10–12 weeks). Treatment intervals are shortened by two weeks if there are any negative changes in clinical parameters. Recent studies of ranibizumab or bevacizumab have shown the TAE regimen to be suitable for the treatment of diabetic MO and MO secondary to CRVO over one year [24, 25].

The objective of this study was to prospectively evaluate the 12-month efficacy and safety of an aflibercept TAE regimen in patients with MO secondary to CRVO.

## 2. Methods

### 2.1. Trial Design

This phase IV, prospective, open-label, single-arm, multicentre trial was conducted in 11 trial sites (tertiary healthcare facilities located in the Basque Country, Castille and León, Catalonia, Madrid, and Valencia autonomous communities) in Spain (NEUTON Trial (RET-AFLI-2014-01); EudraCT Number: 2014-000975-21). The trial was conducted in accordance with the ethical principles of the declaration of Helsinki and Good Clinical Practice guidelines. All ethics committees approved the trial protocol and its amendments.

### 2.2. Patients

The main inclusion criteria were as follows: willingness to provide informed consent; aged ≥18 years; a diagnosis of MO secondary to RCVO within the last six months with mean central subfield thickness of ≥250 *µ*m (spectral-domain optical coherence tomography (SD-OCT)); a baseline BCVA score in the study eye of between 73 and 24 early treatment diabetic retinopathy study (ETDRS) letters (inclusive) measured using the ETDRS chart at four meters (Snellen equivalent: 20/40 to 20/320); a baseline BCVA score of greater ≥20/400 in the Snellen optotype (0.05 decimal, 1 line of sight) in the contralateral eye; the absence of cataracts or other eye diseases that may affect visual acuity.

The main exclusion criteria (in the study eye) were as follows: prior anti-VEGF treatment, photodynamic therapy, corticosteroid treatment or thermal laser treatment; intraocular surgery (including cataract surgery) within three months prior to first aflibercept administration; scarring, fibrosis or atrophy that affects the centre of the fovea; ruptures/tears in the pigmentary retinal epithelial that affect the fovea; severe proliferative macular ischemia or iris rubeosis; prior vitrectomy, submacular surgery or any other surgical procedure for nAMD; or active intraocular inflammation in the study eye.

### 2.3. Trial Interventions

During the loading phase (first three months), IVT aflibercept 2 mg (40 mg/ml) was given once monthly (Week 0, 4, and 8). If the patient improved or showed signs of BCVA stability (no change or a decrease of <5 EDTRS letters during three consecutive evaluations) and/or visual and morphological outcomes (an increase of ≥50 *µ*m central retinal thickness with the absence of new cystic alterations or subretinal fluid or the absence of persistent or recurrent exudation (intraretinal/subretinal) by SD-OCT) after three consecutive visits (Week 4, 8, and 12), the TAE phase began six weeks thereafter (at Week 18). The treatment window was extended by two weeks per visit if no evidence of disease activity was observed (in relation to the period since the last visit) for a maximum of 12 weeks. If there were signs of disease, the patient was retreated and the next visit was scheduled for four weeks later. The trial concluded at the end-of-trial (EOT) visit at Month 12.

### 2.4. Trial Procedures

At baseline eligibility criteria, demographic data, medical history, physical examination, concomitant medication, pregnancy test (if applicable), intraocular pressure (IOP), BCVA score, SD-OCT, fundus fluorescein angiography (FA), retinography, eye fundus photography, and adverse events (AEs) were assessed or reported. During the loading phase (Month 0–2; Week 0–8), TAE phase (Month 3–12; Weeks 12–48), and EOT visit (Month 12; Week 52) concomitant medication, IOP, BCVA, SD-OCT, FA imaging, and AEs were assessed or reported. All FA images and retinographies were sent to the Instituto Oftalmológico Gómez-Ulla reading centre (Responsible reader: Francisco Gómez-Ulla). Optical coherence tomography assessments were performed using Zeiss® (Cirrus version 4.0 or higher), Topcon® (3D, 2000 or 2000 plus), or Heidelberg® (Spectralis version 5.1 or higher) SD-OCT imaging devices, dependent on availability at each trial site. Patients were evaluated with the same device for the duration of the trial.

### 2.5. Trial Outcomes

The primary outcome was mean change in BCVA (ETDRS letters) between baseline and Month 12 (EOT) visit. Secondary outcomes included proportion of patients gaining ≥15 ETDRS letters at Month 12; mean change in BCVA at Month 3 (Week 12–14, after loading doses) versus baseline; mean changes in foveal thicknesses (SD-OCT) at months 3 and 12 versus baseline; proportion of patients free of MO (SD-OCT; central foveal thickness < 200 *µ*m) at Months 3 and 12; mean number of injections; and proportion of patients not requiring additional TAE injections. Safety outcomes included ocular and nonocular AEs and serious AEs (SAEs). Key nonocular adverse events were considered to be those defined by the Antiplatelet Trialists' Collaboration as Arteriothrombolic Events (APTC ATE) [26].

### 2.6. Sample Size and Statistical Analysis

Based on the 1-year results from the GALILEO trial (mean change in BCVA of 16.9 ETDRS letters in patients with MO secondary to CRVO receiving aflibercept every month for 20 weeks followed by PRN thereafter) [19], a total of 43 patients was estimated to detect a significant mean change in BCVA at 12 months with 95% power, a significance level (alpha) of 0.01, and a standard deviation of 25 letters. Estimating a 15% loss to follow-up, a sample size of 50 patients was planned.

The intention-to-treat (ITT) population used for all efficacy analyses included all patients enrolled in the trial who had received at least one dose of aflibercept and who had undergone at least one postbaseline BCVA assessment. The safety population used for all safety analyses included all patients who had received at least one dose of aflibercept.

All data were descriptively analysed. Continuous variables were presented with the number of observations, mean, 95% confidence interval (CI) for the mean, median, standard deviation (SD), and interquartile range (IQR). Categorical variables, however, were described in terms of frequencies and percentages. Changes from baseline were analysed using McNemar's tests for categorical variables and parametric (Student's *t*-test for paired data) or nonparametric (Wilcoxon signed-rank test) tests for continuous variables, as applicable. The level of significance used for all tests was 0.05 (two-tailed). No imputation for missing data was performed.

Data analysis was performed using the SAS® statistical package for Windows (version 9.4, SAS Institute Inc., Cary, U.S.).

## 3. Results

### 3.1. Patient Disposition and Baseline Characteristics

A total of 31 eyes from 31 patients were screened for trial inclusion, and 24 eyes from 24 patients were enrolled in the trial between 23 January 2015 and 17 March 2016. Eighteen (58.6%) patients completed the 12 months of follow-up; two (8.3%) patients discontinued due to a withdrawal of consent, one (4.2%) due to a protocol deviation, one (4.2%) due to a loss of visual acuity without MO, one (4.2%) lost to follow-up, and one (4.2%) withdrawn due to not meeting all eligibility criteria.

Baseline characteristics are shown in Table 1. The mean ± SD age at trial inclusion was 62.8 ± 15.0 years, and 54.2% were male. Median (IQR) time since diagnosis was 7.6 (3.0, 15.2) days with a mean ± SD intraocular pressure (IOP) of 15.7 ± 3.0 mmHg. Arterial hypertension and dyslipidaemia were the most common medical history or concomitant pathologies.

### 3.2. BCVA at Month 12

The mean ± SD baseline BCVAs in the study and contralateral eye were 56.0 ± 16.5 and 81.0 ± 9.3 ETDRS letters, respectively (Tables 1 and 2). At Month 12, mean ± SD BCVA in the study eye was 74.1 ± 17.6, corresponding to a mean (95% CI) increase of 14.8 (8.2, 21.4) ETDRS letters (*P*=0.0001) (Table 2).

### 3.3. Secondary Endpoints

The mean ± SD BCVA after the three monthly aflibercept IVT loading doses was 70.8 ± 19.6, a mean (95% CI) increase of 14.0 (7.8, 20.2) ETDRS letters (*P*=0.0001) (Table 2). Analysis of BCVA over time showed a relatively stable improvement in BCVA after three months that was maintained over follow-up until Month 12 (Table 2). Twelve (50.0%) patients gained ≥15 ETDRS letters by Month 12.

Mean ± SD foveal thickness decreased from 569.4 ± 216.8 *µ*m at baseline to a mean (95% CI) of 291.9 (223.1, 360.7) at Month 3, a significant reduction of 272.2 (167.0, 377.4) *µ*m (*P* < 0.0001) (Table 2). At Month 12, the reduction in foveal thickness was 296.0 (196.8, 395.1) *µ*m (*P* < 0.0001).

Nineteen (79.2%) patients did not exhibit signs of MO at Month 3 or Month 12 (Table 2). One (4.2%) patient continued to exhibit MO for the duration of the study, one (4.2%) patient resolved during the TAE phase, and one (4.2%) patient temporarily resolved during the loading phase but relapsed during the TAE phase.

### 3.4. Exposure to Aflibercept

**Figure 1:**
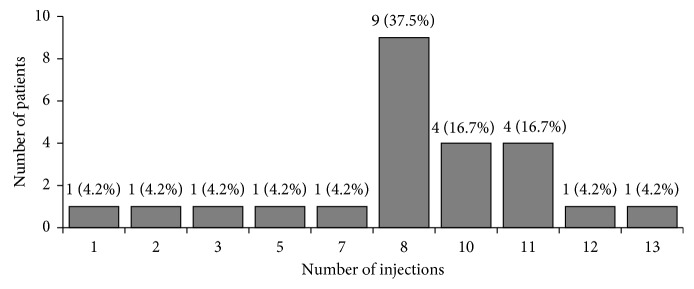
Number of aflibercept injections received by patients during the trial.

Twenty-two (91.7%) patients completed the loading phase (three aflibercept injections); one (4.2%) patient was withdrawn after receiving one injection, and one (4.2%) patient was lost to follow-up after receiving two injections. A mean ± SD of 8.3 ± 3.0 aflibercept injections were received by patients during the trial (Table 2) with 14 (58.3%) receiving ≤8 injections (Figure 1).

### 3.5. Safety

Overall, one (4.2%) patient experienced a nonfatal APTC ATE event (stroke) of moderate severity that was unrelated to study treatment, required hospitalisation prolongation, and was resolved by the end of the study (Table 3). The same patient also experienced a SAE (lung neoplasm surgery) that was of moderate severity, unrelated to study treatment, but required hospitalisation prolongation. Nonserious ocular AEs were experienced by five (20.8%) patients; the most severe AE was vitreous detachment of moderate severity that had not resolved at the end of the study. One (4.2%) patient experienced two ocular AEs (aggravated cataracts and epiretinal membrane), both of mild severity and both not resolved at the end of the study. With regard to systemic AEs, nine (37.5%) patients experienced at least one AE; only one AE (bronchitis) was reported for over one patient—both patients experienced bronchitis of mild severity that was resolved by the end of the study.

## 4. Discussion

Currently, TAE regimens are increasingly used in daily practice for the management of exudative macular diseases to the detriment of fixed and PRN regimens. On one hand, the main advantages of TAE are: (1) proactive treatment that avoids further retinal cells damage derived from disease recurrence, (2) reduction in the number of monitoring visits, (3) predictability of the treatment administration both for the patient and the ophthalmologist, (4) customized treatment for every patient enabling to know the time of the reactivation of the disease. In addition, TAE offers better anatomical and visual results in comparison with PRN regimens and equivalent results with fixed either monthly or bimonthly treatment regimens [21, 27]. However, one of the main concerns that has been raised is that a prolonged TAE could induce an overtreatment with potential unknown side effects if a dry macula is repeatedly injected over time. Likewise, it has not been clearly defined the optimal moment to interrupt the TAE treatment.

The most recommended TAE regimen is based on a gradual two-week extension interval in the absence of disease activity with a maximal extension of 12 weeks [21]. The extension should be initiated after a loading phase consisting of three consecutive monthly injections. Nevertheless, there is no total consensus about this since some ophthalmologists prefer to initiate the extension after the first injection or after a loading phase of two injections. In the present study, we decided to use a loading phase of three injections to try to maximize an initial visual gain that could be maintained with the subsequent TAE during all the study duration.

Confirmatory phase III randomised, controlled trials have shown monthly followed by PRN IVT aflibercept resulting in improved visual and anatomical outcomes in patients with MO secondary to CRVO; however, it remains unknown whether similar improvements could be maintained under a TAE regimen [4, 19, 20]. In this trial, a significant mean 14 ETDRS letter improvement in BCVA was recorded at the end of the loading phase (Month 3) that was followed by a peak BCVA improvement of 14.8 ETDRS letters at the end of the TAE regimen (Month 12). These findings are consistent with the results from the GALILEO (*N*=103) and COPERNICUS (*N*=115) trials which observed peak BCVA improvements of 18.0 and 17.3 ETDRS letters, respectively, after the six monthly aflibercept doses (primary endpoint) that were maintained at 12 months following a PRN regimen (16.9 and 16.2 ETDRS letter improvement, respectively) [19, 20]. Further similarity can be found in the retrospective study by Rahimy et al. where initial gains in BCVA at three months were maintained at 12 months in patients with MO secondary to CRVO treated with IVT bevacizumab or ranibizumab under a TAE regimen [25].

Despite comparable improvements in BCVA, the proportion of patients in whom a vision gain of ≥15 letters was exhibited at Month 12 (*N*=12, 50%) was less than the proportions reported in the GALILEO (*N*=62, 60.2%) and COPERNICUS trials (*N*=115; 55.3%) [19, 20]. Given the small sample size in the present study, it is unclear whether this disparity is significant and clinically relevant. Differences in study design, such as loading phase duration or data analysis (last observation carried forward approach used for data analysis in the two phase III trials vs. no imputation in this study), may account for or contribute to the observed difference.

Anatomical changes in retinal thickness showed a similar pattern of improvement as BCVA, with a significant reduction in foveal thickness occurring after the loading phase that was maintained at Month 12. The mean reduction in central retinal (foveal) thickness at Month 3 (mean: 272.2 *µ*m reduction) was less than the overall 448.6 *µ*m and 457.2 *µ*m reductions observed at the end of the six monthly injections in the GALILEO and COPERNICUS studies, respectively [19, 20]. While the magnitude of the reduction was smaller in the present study, most likely due to the fewer monthly aflibercept injections, in all trials, these anatomical improvements were maintained at Month 12. Further contrast can be observed compared to TAE bevacizumab or ranibizumab, where reductions in central retinal thickness were continual over the course of 12 months, albeit with a final reduction similar to that presented here at Month 3 [25].

Overall, the proportion of patients with MO decreased by 90.5% (*N*=19) by Month 3, with a similar proportion reported at Month 12; one patient exhibited recurrent MO while another resolved during the TAE phase. As only two patients had unresolved MO by the EOT visit, no comparison of baseline characteristics yield insight into possible contributing factors for this observation.

Exposure to aflibercept was measured by the number of injections, duration of treatment, and the proportion of patients requiring further treatment after receiving the loading doses. The mean (SD) injection interval during the TAE phase was 7.0 (2.2) weeks. The overall mean number of injections was similar to the mean number of bevacizumab or ranibizumab injections when administered on a TAE regimen [25]. This contrasts with the GALILEO and COPERNICUS trials where a respective mean number of 11.8 and 9.9 injections were administered during the first year; however, this is likely attributable to the treatment regimen (six monthly doses followed by PRN) [19, 20].

In terms of safety, two SAEs were reported during the study in one patient. The patient experienced a stroke and lung neoplasm surgery, neither of which were related to trial treatment. The absence of ocular SAEs was in contrast to both the GALILEO and COPERNICUS trials, which reported a respective 1.9% (*N*=2) and 3.5% (*N*=4) of patients experienced ocular SAEs when aflibercept was injected monthly (weeks 0–24) and 8.2% (*N*=8) and 2.7% (*N*=3) of patients when aflibercept was injected PRN (weeks 24–52) [19, 20]. Furthermore, no nonserious AEs related to treatment were reported, either systemic or ocular AEs. The proportion of patients who experienced nonserious ocular AEs in this trial was lower than that reported in the GALILEO trial where 54.8% (between weeks 0–24) and 69.1% (weeks 24–52) of patients receiving aflibercept reported at least one ocular AE [19].

The shortcomings of this trial include the small number of patients enrolled and the absence of a control group, which limit any conclusions being drawn.

## 5. Conclusion

The present trial suggests that aflibercept is efficacious in patients with MO secondary to CRVO when treated with a TAE regimen. The low incidence of ocular AEs suggests the TAE regimen may be preferable over other treatment regimens. Studies in larger cohorts of patients with MO secondary to CRVO should be carried out in order to confirm the results of this trial.

## Figures and Tables

**Table 1:** Baseline characteristics.

	ITT population (*N*=24)
*Baseline characteristics*	
Age (years), mean (SD)	62.8 (15.0)
Male, *N* (%)	13 (54.2)
Caucasian ethnicity, *N* (%)	24 (100.0)
Time from MO secondary to CRVO diagnosis (days), median (IQR)	7.6 (3.0, 15.2)
IOP in study eye (mmHg), mean (SD)	15.7 (3.0)
Baseline BCVA score (ETDRS) in contralateral eye, letters, mean (SD)	81.0 (9.3)
Patients with any relevant medical history/concomitant pathology	23 (95.8)
Arterial hypertension	17 (70.8)
Dyslipidaemia	8 (33.3)
Cataracts	5 (20.8)
Anxiety	3 (12.5)
Diabetes mellitus	3 (12.5)
Obesity	3 (12.5)
Others^*∗*^	17 (70.8)

BCVA: best-corrected visual acuity; CRVO: central retinal vein occlusion; ETDRS: early treatment diabetic retinopathy study; IOP: intraocular pressure; IQR: interquartile range; ITT: intention-to-treat; MO: macular oedema; SD: standard deviation. ^*∗*^Conditions experienced by <10% of the patient population.

**Table 2:** BCVA and secondary endpoints after loading doses (month 3), at end of trial (month 12), and aflibercept exposure.

	ITT population (*N*=24)	*P* value^*∗*^
*BCVA score (ETDRS letters)*		
Week 0 (baseline), *N*	24	
Mean (SD) score	56.0 (16.5)	
Month 3, *N*	21	
Mean (SD) score	70.8 (19.6)	
95% CI	61.9, 79.7	
Mean (95% CI) change from baseline	14.0 (7.8, 20.2)	0.0001
Month 12 (EOT), *N*	21	
Mean (SD) score	74.1 (17.6)	
95% CI	66.1, 82.1	
Mean (95% CI) change from baseline	14.8 (8.2, 21.4)	0.0001

*Proportion gaining ≥15 ETDRS letters at EOT,N(%)*		
Yes	12 (50.0)	
No	9 (37.5)	
Not available	3 (12.5)	

*Retinal (foveal) thickness by SD-OCT (μm)*		
Week 0 (baseline), *N*	24	
Mean (SD) score	569.4 (216.8)	
Month 3, *N*	21	
Mean (SD) score	291.9 (151.2)	
95% CI	223.1, 360.7	
Mean (95% CI) change from baseline	−272.2 (−377.4, −167.0)	<0.0001
Month 12 (EOT), *N*	21	
Mean (SD) score	257.4 (48.4)	
95% CI	235.4, 279.5	
Mean (SD) change from baseline	−296.0 (−395.1, −196.8)	<0.0001

*Proportion with MO (SD-OCT),N(%)*		
Week 0 (baseline), *N*	24	
Yes	24 (100.0)	
Month 3, *N*	21	
Yes	2 (8.3)	
No	19 (79.2)	
Month 12 (EOT), *N*	21	
Yes	2 (8.3)	
No	19 (79.2)	

*Exposure: number of aflibercept injections*		
Number of injections		
Mean (SD)	8.3 (3.0)	
Median (IQR)	8.0 (8.0, 10.5)	
TAE injection interval (weeks)		
Mean (SD)	7.0 (2.2)	
Median (IQR)	6.1 (5.2, 9.1)	

BCVA: best-corrected visual acuity; ETDRS: early treatment diabetic retinopathy study; EOT: end of trial; IQR: interquartile range; ITT: intention-to-treat; MO: macular oedema; SD: standard deviation; SD-OCT: spectral-domain optical coherence tomography. TAE: treat-and-extend. ^*∗*^All values compared to baseline using Student's *t*-test for paired samples.

**Table 3:** Summary of adverse events.

	Safety population (*N*=24) *N* (%)
*Serious adverse events (SAEs)*	
*Nonfatal APTC ATE events* (not related to trial treatment)	1 (4.2)
Stroke	1 (4.2)
*Other nonfatal SAEs*	1 (4.2)
Systemic (nonocular; not related to trial treatment)	
Lung neoplasm surgery	1 (4.2)

*Adverse events (AEs)*	
Nonserious ocular AEs (not related to trial treatment)	5 (20.8)
Keratitis	2 (8.3)
Vitreous detachment	2 (8.3)
Aggravated cataract	1 (4.2)
Epiretinal membrane	1 (4.2)
Hyposphagma	1 (4.2)
Increased intraocular pressure	1 (4.2)
Nonserious systemic (nonocular) AEs (not related to trial treatment)^*∗*^	9 (37.5)
Bronchitis	2 (8.3)

APTC ATE: antiplatelet trialists' collaboration arteriothrombolic event. ^*∗*^AEs experienced by >1 patient presented.

## Data Availability

The data used to support the findings of this study are available from the corresponding author upon request.
